# Book Reviews

**Published:** 1902-11

**Authors:** 


CELLULAR TOXINS, OR THE CHEMICAL FACTORS IN THE CAUSATION OF DISEASE. By VICTOR C. VAUGHAN, Ph. D., M. D., Professor of Hygiene and Physiological Chemistry, and FREDERICK G. NOVY, M. D., Junior Professor of Hygiene and Physiological Chemistry in the University of Michigan. New (4th) edition, revised and enlarged. In one 8vo volume of 480 pages, with six illustrations. Lea Brothers & Co., Philadelphia and New York. 1902. [$3.00 net.]

It is difficult to realise that fourteen years—the period that has elapsed since the first edition of this book appeared, under another name, however,—have intervened between the views then announced and those prevailing today concerning the chemistry of infectious diseases. The nature of the change has been so succinctly stated by the authors themselves that we cannot refrain from quoting them:

> When the first edition of this book was written it was believed by those most competent to speak on the subject, that the basic products of bacterial growth constituted the chief factors in the causation of the infectious diseases; but it has been shown by subsequent discoveries that this conception is erroneous, and we now look for the specific bodies among the synthetic substances formed within the cells of the microörganism.

The discovery of essential facts that research has made, constitutes a reason not only for changing the title of the book, but for reconstructing it so thoroughly as to make the present edition almost a new work. The entire study of the subject embraced in the present title is fascinating and, as presented by these authors, becomes more easily mastered than by many is thought to be possible. The etiological classification of diseases given is simple and brings the subject at once before the reader. Working on this basis the student may soon arrive at a clear understanding of the methods of the authors and will thus be enabled to follow them to their conclusions.

In order to appreciate the treatise it should be studied as a whole, but we may be permitted in this cursory notice to point out some of the chapters as possessing special importance. One of these is chapter four in which the bacterial poisons of some of the infectious diseases are considered. Of special interest are the accounts of the germs of diphtheria, tuberculosis, and of suppuration in general. Another chapter of interest is the ninth which deals with immunity. A careful reading of this chapter will serve to remove many doubtful points relating to this important subject, and to set right many misunderstood questions in regard to it. Still another interesting portion of the work is the chapter on food poisoning, a subject with which every physician should become familiar.

It may be remarked that while much of the work is of a technical character, useful especially to the laboratory worker, there is yet a considerable portion that comes within the domain of the clinician and will prove of great use to him in the intelligent treatment of disease, and to the sanitarian and health officer in the prevention of many maladies.

---

The Diseases of the Throat, Nose, and Ear. By Charles P. Grayson, M. D., Lecturer on Laryngology and Rhinology in the Medical Department, University of Pennsylvania; Physician in charge of the department for diseases of the Nose and Throat in the Hospital of the University of Pennsylvania; Laryngologist and Otologist to the Philadelphia Hospital. Octavo, 540 pages with 129 engravings and 8 colored plates. Philadelphia and New York: Lea Brothers & Company. 1902. [Cloth, $3.00 net.]

Laryngology is becoming rich in its literature, many valuable contributions to it in the nature of textbooks having been made within the last few years; also, there has been much in the way of journalistic contributions that is worthy of preservation, so that the student or junior physician need not lack for book instruction. The chief difficulty they are likely to encounter is in the selection of the best, where the supply is so liberal, and the choice must be limited. Not so with the veteran laryngologist, himself perhaps a creator of or a contributor to the literature of his specialty. The special object of review notices should be to aid the former group in the selection of their books. This we will endeavor to do.

Grayson presents a somewhat novel proposition, which he announces as follows:

> In many of the recently published works that deal with diseases of these specialised regions so great a number of remedies will be found, and such a generous variety in the methods of treatment suggested, that they can scarcely fail to prove embarrassing to the younger or less experienced reader. The author has endeavored to eliminate the difficulty of choice by giving under each disease but one plan of treatment—that which he has found to have been most often successful in subduing its symptoms and shortening its duration.

This, in our opinion, is a distinct advantage given this treatise for the group of readers it is especially intended to reach. The advanced laryngologist may not be disposed toward this plan, but he will be equally eager to obtain the book for he will discover that it is one he cannot afford to ignore or go without. The first two chapters will appeal to beginners in a special manner, for they teach the selection and care of instruments and the examination of the patient. These topics are presented so clearly and so simply that they cannot fail to attract attention.

Another strong feature of the work is the stress laid upon the anatomy and physiology of the nasal structures, and especially does Grayson clearly differentiate the turbinates, which every laryngologist must learn at the threshhold of his career. Turning to the chapter on the ear, the same remark as to the anatomy and physiology section might be made with appropriateness. Again, this author devotes much space to and gives clear and scientific teaching regarding that important and quite common affection, chronic catarrhal otitis media. It is from neglect either to recognise or to give heed early to this disease that many obstinate or incurable maladies later balk or thwart the best skill.

This book is double-leaded in the text from the first chapter to the last, which makes it pleasant reading for the eye; and the finely finished paper permits the illustrations to be clearly brought out, many of the latter being worthy of note. If we mistake not Grayson's laryngology is destined to take a foremost place.

---

American Edition of Nothnagel's Encyclopedia, Vol. III. Diphtheria. By Wm. P. Northrup, M. D., of New York. Measles, Scarlet Fever and German Measles. By Professor Dr. Th. von Jürgensen, Professor of Medicine in the University of Tübingen. Edited, with additions, by William P. Northrup, M. D., Professor of Pediatrics in the University and Bellevue Medical College, New York. Octavo, 672 pages, illustrated, including 24 full-page plates, three of which are in colors. Philadelphia and London: W. B. Saunders & Co. 1902. [Cloth, $5.00 net; half-morocco, $6.00 net.]

Owing to the fact that the German author of the article on diphtheria in the original work had arranged to issue a separate translation of it, the editor of the American edition, Dr. Stengel, was obliged to cast about for an author to write an entirely new article. Fortunately, a most competent one was found in the person of Dr. William P. Northrup, who has presented the subject so completely that no loss is sustained by the numerous readers of the work.

The whole subject of diphtheria is detailed in a most attractive as well as instructive fashion, and especially is the treatment of this direful affliction delineated in a thoroughly scientific manner. The two recent additions to the armamentarium—antitoxin and intubation—are described with much care and their values adequately fixed. Especially is intubation given important place and its technic minutely described. Dr. O'Dwyer's great work in placing intubation upon an enduring footing is properly set forth. Dr. Northrup, having been associated with Dr. O'Dwyer at every step in the perfection of intubation tubes, is particularly fitted to describe this aspect of the treatment of diphtheria.

It is with much satisfaction that we note that Jürgensen has written the article on the acute exanthemata,—measles, scarlet fever and German measles. His distinction as a writer and teacher on internal medicine has been acknowledged for a quarter of a century, indeed since his famous contribution to Ziemssen's Cyclopedia. His monograph on measles in this book will be found comprehensive and is the best exposition of the subject thus far made. So, too, it may be said of that on scarlatina, which is equally comprehensive in clinical details, while for exactitude of statement and clearness of expression it is unrivaled. His discussion of Watson Williams's "fourth disease" will be found interesting. Jürgensen seems to accord it an appropriate place.

The illustrations deserve mention, there being cuts in the text, plates, and color engravings, plate 7, comparing diphtheria of the pharynx and tonsils with follicular tonsillitis, being worthy of special note. The entire book is a creditable companion to the volumes of the series that have preceded it.

---

Applied Surgical Anatomy Regionally Presented, for the use of Students and Practitioners of Medicine. By George Woolsey, A. B., M. D., Professor of Anatomy and Clinical Surgery in the Cornell University Medical College; Surgeon to Bellevue Hospital, New York. Octavo, 511 pages with 125 illustrations. Philadelphia and New York: Lea Brothers & Company. 1902. [Cloth, $5.00 net; leather, $6.00 net.]

The older anatomists, Vesalius and his followers, were wont to infuse the study with something of poetry, something of art as well as science; at least they made artists and the artistic contribute to its interest. The teachers of later days omitted almost everything from their anatomic courses, except the mere description of the tissues, hard and soft, and the viscera. In the closing decade or two of the last century, however, the artistic again began to play an important part in teaching what to many would otherwise prove a dry subject.

Woolsey has adopted this method and has imparted zest to its investigation, by coupling anatomy and surgery, a knowledge of the former in all its details being necessary to those who practise the latter. But it is not alone to surgeons that anatomy must be known, for the physician, likewise, must be acquainted with its mysteries and intricacies. This knowledge can be acquired more readily region by region than in any other way, hence anatomy regionally applied is quite as valuable to him as to the surgeon. Moreover, the undergraduate will find this method of application more interesting as well as more useful.

The author proves himself a practical anatomist by the manner in which he takes up the subject, as well as by the detail with which he applies it through the several regions of the body. The text is clear from contents to index, the sections are well defined by captions in heavy type, and the illustrations are singularly artistic in execution.

Woolsey's anatomy is one of the books that is likely to attract the attention of teachers, practitioners and pupils, because of its value as a textbook, as a work of reference, and as a practical manual for the anatomical laboratory. It is a decided addition to the literature of anatomy because it presents the subject in a different manner from that in general and, further, because it presents it well.

---

PROGRESSIVE MEDICINE. Volume III, September, 1902. A Quarterly Digest of Advances, Discoveries and Improvements in the Medical and Surgical Sciences. Edited by HOBART AMORY HARE, M. D., Professor of Therapeutics and Materia Medica in the Jefferson Medical College of Philadelphia. Octavo, pp. 421, 26 illustrations. Philadelphia and New York: Lea Brothers & Co. [Per annum, in four cloth-bound volumes, $10.00. Per volume, $2.50, by express prepaid to any address.]

In this volume we find considered first, diseases of the thorax and its viscera, including the heart, lungs, and bloodvessels, prepared by William Ewart. The material in this section is of the very best on the important diseases which it embraces. Every physician whose practice includes this field should be familiar with the latest thought on the subject, and here it will be found. The next topic is dermatology and syphilis prepared by William S. Gottheil, who has grouped the important advances and novelties in this branch.

The section on diseases of the nervous system which next follows has been arranged by William G. Spiller. It is full of interesting and instructive suggestions, and records the progress that is making in this great subdivision of medicine. In the section of obstetrics, which is the last considered in the book, Richard C. Norris has compassed the entire territory which this branch deals with. Pregnancy, the management of labor, obstetrical surgery, tumors complicating pregnancy, labor obstructed by pelvic deformity, placenta previa, postpartum hemorrhage, the management of puerperium and the care of the newborn infant, have all been discoursed upon, and everything of value discovered in recent literature has been gleaned and stored in these pages. The entire volume is a credit to all who have participated in its proceedings.

---

THE PRACTICAL MEDICINE SERIES OF YEAR BOOKS. Ten volumes. Issued monthly under the general editorial charge of Gustavus P. Head, M. D., Professor of Laryngology and Rhinology in the Chicago Post-Graduate Medical School. Volume VII. Materia Medica and Therapeutics; Preventive Medicine; Climatology; Forensic Medicine. By GEORGE F. BUTLER, Ph. G., M. D., HENRY B. FAVILL, A. B., M. D., NORMAN BRIDGE, A B., M. D., HAROLD N. MOYER, M. D. 12mo, pp. 277. Chicago: The Year Book Publishers. 1902. [$1 50. Entire series, $7.50.]

In materia medica the newer drugs and remedies are described or referred to in more or less detail, and newer fields of usefulness of older ones are particularly alluded to. Therapeutics is given ample place and much valuable information can be obtained in this book concerning the clinical application of curative resources. These two subjects take up more than half the book.

Preventive medicine has been abstracted to good purpose and the more recent contributions of value noted. The scientific papers of the year relating to climatology have been condensed and herein noted, and the more salient features, bearing on forensic medicine that the year has evolved have been appropriately referred to. In alluding to the trial of the assassin of President McKinley, which the editor says brought out comparatively little of forensic importance, Dr. J. W. Putnam's statement as to the condition of the prisoner is given, and the editor expresses the opinion that there was nothing to show that the assassin was insane, but ventures the suggestion that he belonged to the degenerate class.

The book contains much of value in the several departments, being well worth its price.

---

MASSAGE AND THE ORIGINAL SWEDISH MOVEMENTS. Their Application to Various Diseases of the Body. Lectures before the Training Schools for Nurses connected with the Hospital of the University of Pennsylvania, German Hospital and others of Philadelphia. By KURRE W. OSTROM, from the Royal University of Upsala, Sweden. Fifth edition, revised and enlarged; 115 illustrations. Philadelphia: P. Blakiston's Son & Company. 1902. [Price, $1.00 net.]

For the past twelve years this little work has been the standard reference book on the subjects of which it treats. Ostrom is an acknowledged authority upon massage and an instructor of intelligence upon everything relating to it.

The experience which the author has acquired since the first edition was issued, has enabled him to make this one more valuable, so that now it is as near complete as it is possible to make it. It is of value to the physician as well as the nurse, to the masseuse and masseur. New material has been added in each successive edition and the book is without a rival in its special field.

---

A TEXTBOOK OF PRACTICAL THERAPEUTICS. With especial Reference to the Application of Remedial Measures to Disease and their Employment upon a Rational Basis. By HOBART AMORY HARE, M. D., Professor of Therapeutics and Materia Medica in the Jefferson Medical College of Philadelphia. Ninth edition. Octavo, 851 pages, 105 engravings and four colored plates. Philadelphia and New York: Lea Brothers & Company. 1902. [Cloth, $4.00; leather, $5.00; half-morocco, $5.50 net.]

The editions of this textbook appear in such rapid succession that it causes one to wonder what became of all the books. On a moment's reflection, however, the answer is readily found in the fact that it is just the book the active physician most needs. He generally needs the information he seeks quickly, too, and here he finds it, accessible, clear and adequate. On every occasion we have consulted its pages, and they are many, we have never turned away with disappointment. We have found what we wanted to know and have found it without delay. This means that the book is so well arranged that its contents become readily and easily searched. This, in turn, means a saving of time and a freedom from mental irritation,—both important in these days of work and worry and hurry.

One great advantage in these rapidly appearing editions is the opportunity afforded for revision. The changes in methods of treatment are frequent on account of discoveries that are constantly making in the laboratory and in the clinic room, hence it is important that a treatise like this should keep pace with advances, and record the very latest that scientific research produces. This edition shows a thorough revision of the text, an addition of every new measure of value, and nearly one hundred new illustrations. The prefaces are dated, a convenience which is appreciated by all who wish to trace the editions through their several dates of publication. This must continue to be the textbook *par excellence* of therapeutics.

---

The Roller Bandage. By William Barton Hopkins, surgeon to Pennsylvania Hospital and to the Orthopedic Hospital and Infirmary for Nervous Diseases. 12mo, pp 178. Fifth edition, revised. Philadelphia: J. B. Lippincott Company. 1902. [Price, $1.50]

To become proficient in the art of bandaging requires much practice; indeed, it may be added that a mechanical aptitude for the work is also needed. A skilful bandager must know where to begin and where to end, how snug to draw, or how loose to lay the bandage. Moreover, the texture of the bandage must be adapted to the particular condition in hand, and the operator must possess expert knowledge as to the precise length and width required therefor. These and many other facts must be compassed by the expert bandager.

In this book, in text and by illustration, almost the last word has been said on the application of the roller bandage to the several parts of the body. It teaches how to prepare it as well as how to apply it, and all that can be learned from a book is most felicitously presented to the student of the subject. The bandages have been applied to a living model and photographs taken during the application at some stage or when completed and from these reproductions the plates for this edition were made. The electrotypes and plates of the preceding edition were destroyed by fire, hence this one has been entirely remodeled and new engravings have been made for it. It is a model of conciseness and perfection.

---

The Practitioner's Manual. A Condensed System of General Medical Diagnosis and Treatment. By Charles Warrenne Allen, M. D, Consulting Genitourinary Surgeon to the City (Charity) Hospital, Consulting Dermatologist to the Randall's Island Hospital, to the Hackensack Hospital, etc. Octavo, pp. 893. Second edition, revised and enlarged. New York: William Wood & Company. 1902. [Price, muslin, $6.00; half-morocco, $7.00 net.]

The many excellent features of this work have been the subject of discussion heretofore in these columns. It is a book intended chiefly for the "busy practitioner," and in this aim it appears to have hit the mark for the first edition—a large one—has become exhausted in comparatively a short time. The prefaces are not dated, which is a fashion or a fad to be condemned. Nothing can be gained by such a weak omission except to annoy the reviewer; and we are not sure that this is a gain. However, we find on reference to the indices of the JOURNAL that the first edition was published in 1898 and was noticed in the magazine for February, 1899. What we then said about the book is equally true now, with even more emphasis.

The profession of medicine is over-stocked with annuals, yearbooks, and reference volumes, but this one, constructed on new lines, has a place. It gives the searcher quick information of a reliable and recent kind on subjects of immediate value at the bedside. No physician can carry all the clinical data in his head that are required in his daily rounds attending to an exacting practice, but here he can get information to reinforce at once his stock of knowledge. He can obtain hints upon every subject from "abscesses of the brain" to vertigo,"—the subjects that begin and end the volume. An appendix furnishes a table of equivalents and methods of converting apothecaries' weights and measures into the decimal system. An excellent, comprehensive index closes the book.

---

INTERNATIONAL CLINICS. A Quarterly of Illustrated Clinical Lectures and especially prepared Articles on Medicine, Neurology, Surgery, Therapeutics, Obstetrics, Pediatrics, Pathology, Dermatology, Diseases of the Eye, Ear, Nose and Throat, and other Topics of Interest to Students and Practitioners by leading Members of the Medical Profession throughout the World. Edited by HENRY W. CATTELL, A. M., M. D., Philadelphia, U. S. A., with the Collaboration of John B. Murphy, M D., Chicago; Alexander D. Blackader, M. D., Montreal; H. C. Wood, M. D., Philadelphia; T. M. Rotch, M. D., Boston; E. Landolt, M. D., Paris; Thomas G. Morton, M. D., Philadelphia; James J. Walsh, M. D., New York; J. W. Ballantyne, M. D., Edinburgh, and John Harold, M. D., London, with Regular Correspondents in Montreal, London, Paris, Leipsic and Vienna. Volume II. Twelfth series. Philadelphia and London: J. B. Lippincott Company. 1902. [Cloth, $2.co each.]

The departments considered in this volume are therapeutics, medicine, surgery, obstetrics and gynecology. In the first there are seven lectures by foreign teachers, to which are added a few pages of selected prescriptions which will prove of benefit to the younger physicians. In the medicine section there are ten lectures by American and foreign teachers, elaborating many important clinical observations not dealt with adequately in the textbooks.

The Surgery section also is replete with valuable thought, though there are in it but five lectures. Obstetrics and gynecology is the weakest section in the book, but that is made up for by other interesting material. A biographical sketch of Dr. John B. Murphy, of Chicago, accompanied by a splendid portrait of this distinguished surgeon will be read with satisfaction by his many admirers, who may here learn important hints as to the conduct of the business side of professional work.

Three special articles follow, one giving an outline of the organisation and work of the medical department of the army, one giving hints upon the management of private hospitals, and another on the function of the digestive glands. The illustrations are better and more numerous than usual.

A MANUAL OF INSTRUCTION IN THE PRINCIPLES OF PROMPT AID TO THE INJURED. Including a Chapter on Hygiene and the Drill Regulations for the Hospital Corps, U. S. A. Designed for Military and Civil Use. By ALVAH H. DOTY, M. D., Health Officer of the Port of New York. Fourth edition, revised and enlarged. New York; D. Appleton & Co. 1902.

In these days the laity has been taught much that pertains to early succor in case of injury or sudden illness, the object being a humane one, namely to afford temporary relief pending the arrival of a physician. It is important that such instruction should be based upon sound principles and at the same time be not so technical as to thwart its purpose. Dr. Doty's Manual has done much to reduce this teaching to its proper sphere and this fourth edition gives token of an appreciation of the good work the author is accomplishing. It teaches just enough of anatomy and physiology to ensure an intellignt application of the principles of prompt aid to the injured and distressed, and the text presents so clearly the points sought to be enforced that it makes the book the most approved source of information in its class. It is as important for the nurse, the ambulance officer, the interne, and the emergency physician as it is for those of the laity who would possess intelligence on this subject with which it deals. Indeed, it is a good book to find in every well regulated household.

